# Rehabilitation Program for Gait Training Using UAN.GO, a Powered Exoskeleton: A Case Report

**DOI:** 10.3390/neurolint14020043

**Published:** 2022-06-16

**Authors:** Gianfranco Lamberti, Gianluca Sesenna, Qamil Paja, Gianluca Ciardi

**Affiliations:** 1 Spinal Unit, Azienda USL, 29121 Piacenza, Italy; g.lamberti2@ausl.pc.it; 2 Degree Course of Physiotherapy, Piacenza Training Center, University of Parma, 29017 Fiorenzuola d’Arda, Italy; 3 U&O, 29017 Fiorenzuola d’Arda, Italy; gianluca.sesenna@uando.it; 4 Degree Course of Physiotherapy Student, Piacenza Training Center, University of Parma, 29017 Fiorenzuola d’Arda, Italy; pajaqamil@gmail.com

**Keywords:** spinal cord injury, robotic exoskeleton, rehabilitation, walking

## Abstract

Background: Spinal cord injury is characterized by the interruption of neural pathways of the spinal cord, with alteration of sensory, motor, and autonomic functions. Robotic-assisted gait training offers many possibilities, including the capability to reach a physiological gait pattern. Methods: A training protocol with UAN.GO^®^, an active lower limb exoskeleton, was developed. A participant having D10 complete SCI was recruited for this study. The training protocol was composed by 13 sessions, lasting 1.5 h each. The effectiveness of the protocol was evaluated through the mobility performance during the 6 MWT, the level of exertion perceived administrating Borg RPE at the end of each 6 MWT. Furthermore, time and effort required by the participant to earn a higher level of skills were considered. Results: A significant improvement was registered in the six MWT (t_0_ = 45.64 m t_1_ = 84.87 m). Data referring to the mean level of exertion remained stable. The patient successfully achieved a higher level of independence and functional mobility with the exoskeleton. Discussion: The findings from this preliminary study suggest that UAN.GO can be a valid tool for walking rehabilitation of spinal cord injury patients, allowing the achievement of greater mobility performances.

## 1. Introduction

Spinal cord injury (SCI) is a neurological condition that produces devastating effects on affected people, due to permanent disabilities resulting also in heavy implications in terms of socioeconomic and health care system impact [1]. Between 250,000 and 500,000 patients every year suffer a SCI, and most of these cases are traumatic and due to preventable causes such as motor vehicle accidents [2].

The interruption of neural pathways of the spinal cord causes a very large range of clinical outcomes depending on severity, extension and level of injury, with partial or complete loss of sensory and motor function below neurological level [3]. Often the autonomous system is involved too, compromising the cardiopulmonary system, urogenital and bowel functions [4,5].

Walking impairment is one of the major disabilities caused by a SCI; it causes an additional reduction in voluntary mobility, forcing individuals to use wheelchairs as preferential way to move, and causing secondary damages such as pressure ulcers, osteoporosis, and muscle atrophy and retractions [6]. Furthermore, patients with SCI are at a higher risk for developing psychological disorders such as depression, anxiety and PTSD (post- traumatic stress disorder), leading to worse quality of life [7].

The rehabilitation can be organized through three phases: acute, subacute, and chronic. The first two phases generally correspond to the natural history of neuro-recovery, being between 12- and 18-months post injury [8]. In this period, rehabilitation focuses on preventing secondary complications, promoting neuroregeneration and optimizing function to obtain the highest long-term level of independence and health. In the chronic phase, once that recovery is stabilized, treatment focuses on compensatory or assistive approaches. A multidisciplinary approach, as underlined in literature, is critical in any phase of SCI rehabilitation [9,10]. The increase in neural activity is training-dependent; a physiotherapy program focused on high-repetition exercises is fundamental to allow new movements and compensatory strategies learning [9].

A relatively recent technological progress concerns robotic exoskeletons, which allow individuals with gait impairment to experience walking again [11]. Exoskeletons consist in wearable devices used to increase or substitute, when compromised, human limbs functionality [12]. This technology, used by many industries to replace human labor and carry out highly repetitive or heavy tasks, is also used to enable individuals with SCI to reach orthostatic position and walk, providing a high intensity and reproducible lower limb movement in a more physiological way than traditional physiotherapy [11,13,14]. Individuals undergoing intensive rehabilitation plans involving robotic-assisted gait training (RAGT) show a better clinical condition, due to reorganization at the spinal cord level [14,15].

Rehabilitation with wearable robotic exoskeletons reduces the risk of secondary complications, improves the cardio-pulmonary and visceral functionality, reduces pain and spasticity while the need for assistance by therapists is reduced and the duration of the training is increased [16,17]. Despite the difference among devices currently available, the field of exoskeleton rehabilitation for spinal cord injury patients is still strongly growing, and several works have already shown scientific evidence of improved functional capacity [18,19,20], functional abilities [18] patient’s satisfaction and expectations [19,21], overall cardio-pulmonary compliance [22], trunk and lower limb performance [23,24,25], and gait performance [18,19,20,21,22,23,24,25,26].

The aim of the present report is to develop and apply a training protocol with the exoskeleton for lower limbs, UAN.GO^®^. The defined protocol was applied with a patient suffering from complete SCI.

## 2. Materials and Methods

Based on what has been reported in the literature [12,27,28,29,30,31,32,33,34,35], a specific training protocol was developed for the realization of this study, in order to set a standard in the conduct of a rehabilitation path with UAN.GO^®^, the lower limb robotic exoskeleton designed and produced by U&O Technologies s.r.l., an Italian MedTech company that designs, manufactures and markets innovative medical exoskeletons and aids for the rehabilitation of people with gait disorders.

A single patient voluntarily applied for the conduct of the study. Inclusion criteria were: adult with SCI, weight less than 100 kg, height from 160 cm to 190 cm. Exclusion criteria: severe spasticity (Ashworth scale scoring > 3), heterotopic ossification or anything limiting ROM, cognitive or psychiatric disorders that can interfere with the conduct of the training. Table 1 shows the inclusion criteria that the patient has to meet during the preliminary meeting in order to begin the training.

The patient, a 29-years-old male at the time of the conduction of this study affected with SCI level D10, Asia A [21] since 2015, was enrolled after signing informed consent. The patient underwent a preliminary session necessary to assess the eligibility of the treatment and the anthropometric compatibility with UAN.GO^®^. The training consisted of 13 sessions, lasting about 1.5 h and performed once a week. No ethics committee approval was required, as suggested for clinical case reports by the Aven guidelines [31].

At the end of each session, six MWT [32] and BORG scale [33] were monitored. To assess the effectiveness of the training program, time and effort required to achieve the ability to walk and gain speed were registered in terms of number of training sessions comparing them with the benchmark of other devices.

Data were collected at the beginning of the training (T_0_) and at the end (T_1_).

UAN.GO^®^ exoskeleton is a powered lower limb exoskeleton designed by U&O Technologies srl. In 2021 UAN.GO^®^ was certified as a medical device CE IIA in accordance with the 93/42 CEE directives for personal and clinical use. UAN.GO^®^ allows people with lower limbs and mobility impairments to walk again in a physiological way through an overground gait training. The device is provided with four motorized joints (hips and knees) and four passive joints (ankles and feet), coordinated by a cutting-edge software system and supported by sophisticated movement sensors. This eight-joint system contributes to the definition of an optimized gait timing and kinematics while the weight is completely discharged to the ground. 

**Figure 1:**
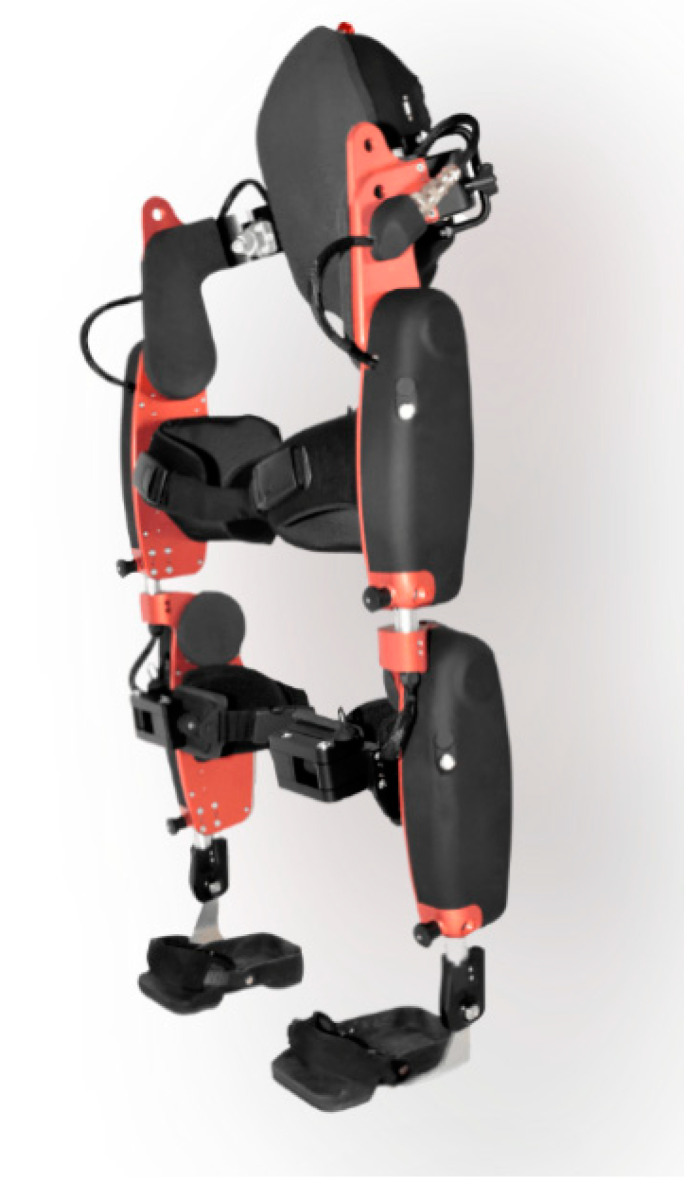
Sagittal view of UAN.GO, the exoskeleton used for the present case report. The device is provided of a bilateral four-joint system (hip, knee, ankle and foot), in which proximal joint are computerized (hip and knee), distal ones are fixed (ankle and foot). A central unit allows movement maps execution.

In Figure 1 a frontal view of UAN.GO^®^ exoskeleton; it needs only 2 min to reach anatomical configuration on patient’s size; the device allows the patient to stand up, sit down, walk, perform steps and stairs, with the possibility to customize many maps in accordance with the patient’s skill level.

The “assisted mode” is managed by the therapist or the caregiver, that selects the movement from the onboard touch screen on the back, and activates each action through “start and stop” buttons; setting “autonomous mode” allows the patient to activate each movement through his trunk.

Passive or active-assisted can be set according to the level of strength requested to the device.

UAN.GO^®^ is intended for individuals with complete SCI from T4 to L5, or incomplete SCI from C7 with preserved upper extremity motor function of at least 4/5 MRC in both arms. The device is also designed in patients with gait impairment by acute/degenerative disorders such as stroke, multiple sclerosis or Parkinson’s disease.

**Figure 2:**
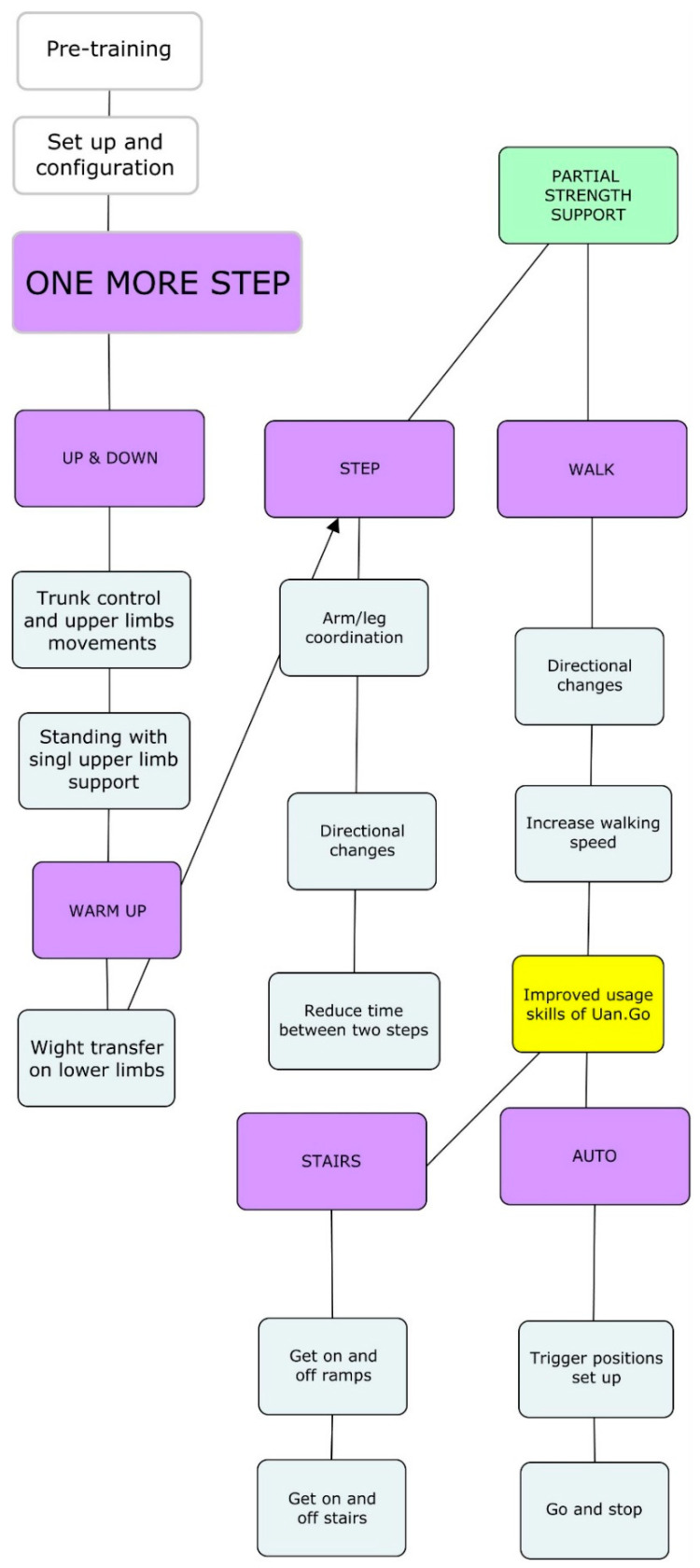
ONE MORE STEP is the treatment protocol developed for this study.

Figure 2 shows the treatment protocol, named “ONE MORE STEP”, developed for this study. The pre-training phase consists in the explanation of the study, the preliminary interview and assessment, the setting of the exoskeleton, the dressing and ‘Up and Down’ to allow the patient a first approach to the device. The real training starts with the learning of the ability of standing up and sitting down and the control of the upright position (UP&DOWN). Next, the patient begins to move the lower limbs, learning how weight transfers from one leg to the other through walking in place (WARM UP). Once the patient learns loading his weight and move his trunk, he can start to walk with the STEP maps; caregiver activates one step at a time once the patient reaches the right position with his trunk. With STEP maps, the patient learns to better coordinate upper and lower limbs in order to WALK with the same maps and speeds learned. Lastly, training is completed by STAIRS skills, using the device in “autonomous mode” (AUTO). The patient has learned how to control the weight transfer through the trunk in order to reach a previously defined point in space set on the device that allow to start and stop walking without the need for the caregiver to push any button. 

What is recommended through the duration of the whole training is as follows:

- To not force the patient to achieve skills that he is not interested in
- To respect patient’s own learning time
- To aim at an improvement of the patient’s quality of life, not only in physical terms but also psychologically and socially.

## 3. Results

**Figure 3:**
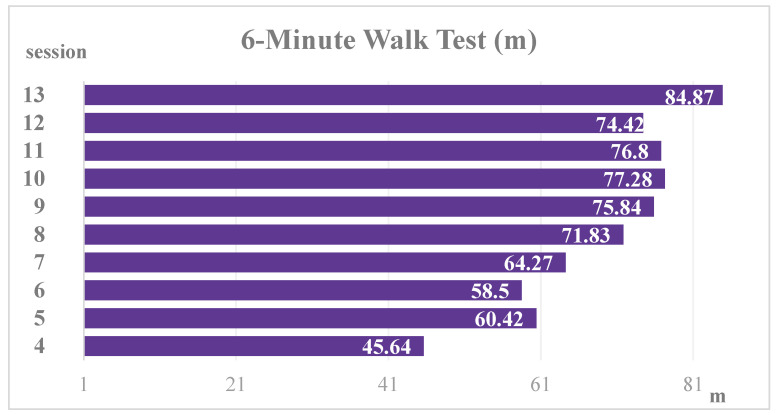
Trend of six MWT.

The training ended at the 13th session without any adverse event occurring. A noteworthy improvement was registered in the motor performance during the 6 MWT, that shows a gradual increase of the walked distance as shown in Figure 3. The distance going from 45 m to 85 m, with an improvement of 40 m overcomes what Bohannon et al. [34] have set as the minimum value for clinical significance considered between 14 m and 30.5 m.

**Figure 4:**
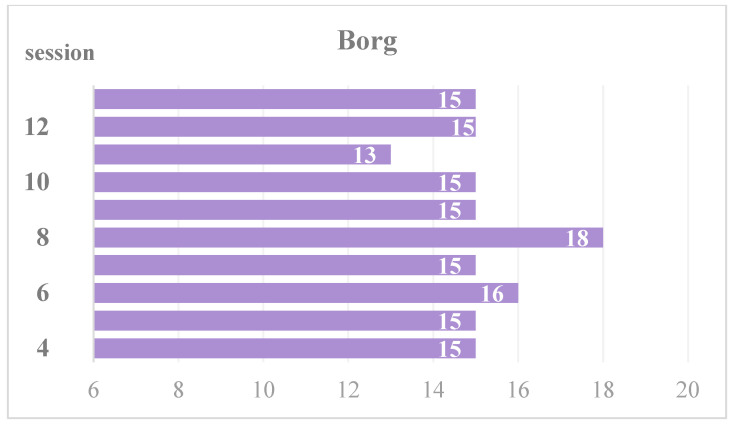
Trend of the BORG score.

Figure 4 shows data referring to the exertion perceived at the end of each 6 MWT assessed through the administration of the BORG RPE Scale and revealing a steady trend. The result can be considered coherent since, retaining exertion, the distance, and the complexity of gait patterns (wider ROMs and angular speed of movement) have increased.

**Scheme 1:**
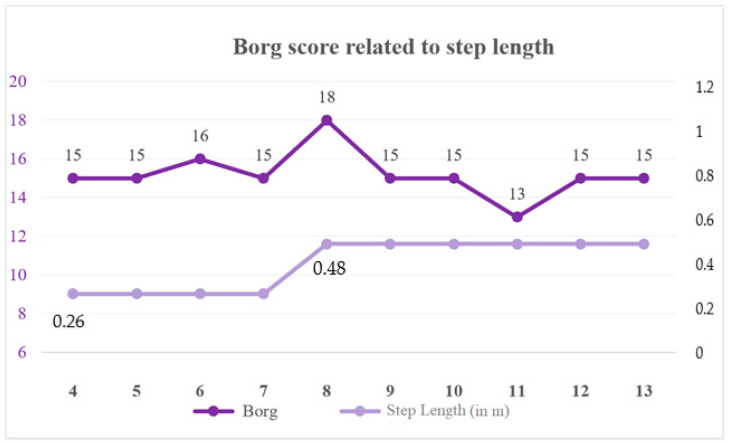
Relation between the Borg score and the length of the step of the movement map used during the session.

Regarding the level of independence and skill reached by the patient, at the last training session he could stand up and sit down in the “autonomous mode” just with supervision. The patient could perform up to 50 consecutive steps (in STEP mode) with supervision only in the “autonomous mode”. For the WALK, the patient needs minimum assistance with contact only for long distances. Scheme 1 shows the relationship between the exertion perceived during the six MWT and the step length. From the fourth session, the step was 0.26 m long and from the eighth session the patient could manage a 0.48 m long step for the entire duration of the six MWT. Table 2 shows the skills achieved during each session considering the independence in the dressing of UAN.GO^®^ too. 

## 4. Discussion

The present study was compliant with CARE guidelines [35].

The aim of the present study was to define and apply a standardized training protocol with a powered lower limb exoskeleton with a participant affected with SCI and verify the eventual skill gaining.

The protocol was well tolerated by the participant and, at the same time, the therapists’ number and physical fatigue were reduced with respect to the traditional physiotherapy [14].

Data referring to perceived exertion emerging from the administration of the BORG scale are coherent with the increase of the walked distance during each six MWT performed. A peak of the Borg can be noted during the eighth session when the patient used for the first time during the six MWT a gait map that allows a step length of 0.48 m while he always used a map of 0.26 m step length. It must be also considered that the patient reported fatigue mainly due to upper limbs overload, and literature states that with continuous training this aspect improves too [27].

Furthermore, at the end of the training, the patient was able to walk with a 0.64 m step length map but only for reduced periods of time. A larger number of sessions could be useful to assess a possible further improvement.

**Figure 5:**
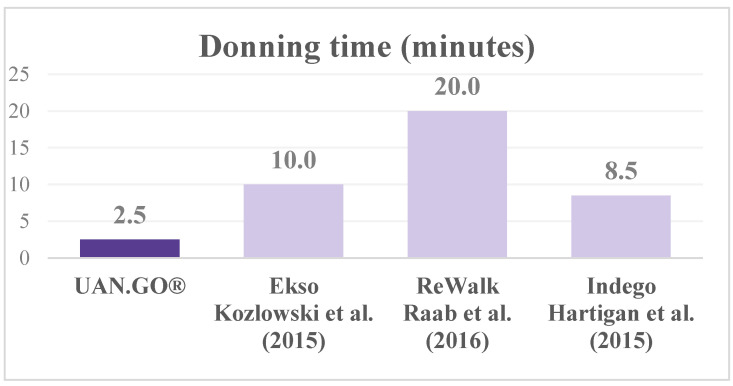
Donning times comparison between Uan.Go, Ekso [36], ReWalk [37] and Indego [38] exoskeletons.

A specific discussion is needed about the skill to dress the exoskeleton, which is reported in literature as significance index of compliance and feasibility of the protocol [36,37,38]. In our experience, from the third session, the patient was able to don and doff the exoskeleton in 2 min independently (with the exception of the feet bands for which he required assistance) allowing increase training time. Figure 5 shows the time required to don with other powered lower limbs exoskeletons [36,37,38].

With respect to other studies that assess the skill gain with other exoskeletons, participants using Ekso^®^ needed a moderate level of assistance even during the sixth session for donning and doffing [39] while the level of independence reached with ReWalk is comparable to that reached by the participant of this study [40]. The setting of the size of UAN.GO^®^ is carried out in 30 s by the therapist or the caregiver just before the donning.

After four sessions, the participant was able to stand up and sit down just with supervision.

It took four sessions for the participant to be so proficient in the walk to be able to complete a six MWT. Between the first six MWT (45 m) and the second test (60 m) there’s an important improvement that can be imputed to the effect of first administration of a test not known by the participant [41]. The improvement between the second and the last six MWT still remains significant with respect to the minimal clinically important difference for change in six MWT distance defined by Bohannon et al. [34].

The speed reached at the end of the training was 0.24 m/s, two times the starting speed during the first six MWT. The reached speed in this case is compliant with what reported in the literature for patients with SCI who don’t walk [42].

In general, not being reported adverse events and being remarkable the skill gain and the independence reached by the participant, the training with UAN.GO^®^ can be a useful tool to be implemented in the treatment of patients affected by SCI. A final consideration is needed about prediction of skills acquiring during exoskeleton training: recent studies highlighted some patient’s characteristics as predictors of good training result [43,44,45]. Particularly, literature describes level of injury as the best predictor in the first skill acquiring phase (4 weeks according to van Dijsseldonk et al., 43), while by the fifth week to the end of program low BMI, higher physical activity levels and low age are most influencing variables [44]. According to this perspective, our patient had some good skill predictors in his history: lower level of injury (D10), young age (29 at the moment of the study), physical activity (he played wheelchair basketball); final results was so in line with literature suggestion. We can hypnotize that UAN.GO exoskeleton follows previous studied prediction models, but since its difference by other device in terms of weight, support over the ground future studies are needed to better clarify this aspect. As for predictors not depending by the patient, the meta-analysis of Miller et al. [45] state that an adequate time of skill-training (more than six sessions), learning about safety and space management during exoskeleton use could improve patient ability to walk without assistance. In the present case report the patient began able to manage only in supervision the exoskeleton at the end of the training (13 sessions), walking in a well-known and safe environment. These results, again, were in line with previous studies findings.

However, since this study is a case report and having no statistical value, it can only be considered as a first step in the validation of the ONE MORE STEP protocol. Moreover, the large variety in terms of clinical manifestations that the SCI represents, reveal the necessity of further studies that could assess the protocol developed for this study in terms of the SCI level, extension of the injury, and also in terms of motivation and psychosocial aspects of the participants.

## 5. Conclusions

Physiotherapy treatment including RAGT with UAN.GO^®^ allows individuals with complete thoracic SCI to walk again. The “ONE MORE STEP” protocol seems to be an effective and useful tool in the approach and definition of personalized training paths with powered lower limbs exoskeletons. Since this study has no statistical value and represents just a pilot on this type of treatments, the protocol should be applied on a larger scale with more participants and a variety of clinical and functional stages, in order to assess an eventual relation between the level of injury and the gain of skill.

## Figures and Tables

**Table 1:** Inclusion criteria for training with UAN.GO^®^.

Inclusion Criteria	Exclusion Criteria
Lower limbs weakness or paralysis	Heterotopic ossifications limiting ROM
The ability to understand and sign the	Unhealed fractures or instable vertebral
informed consent and the liability waiver	column
Absence of functional ROM limitations	Persistent orthostatic hypotension
Ashworth Scale lower or equal to 3	Uncontrolled autonomic dysreflexia
Upper limbs preservation and ability to use a walker or crutches	Skin integrity issues
Ability to follow directions	Hospitalization during the last 3 months due to heart attack, heart surgery acute earth failure
Preserved learning ability	Cognitive or psychiatric disorders that can interfere with the conduct of the training
Height between 160 cm and 195 cm	Significant osteoporosis
Weight lower than 100 kg	Pregnancy

**Table 2:** Skills per session.

	Skill			Session
**Dressing**	Donning			1
	Doffing			1
	Fastening tibial bands			2
	Fastening femoral bands			2
	Fastening abdomnial band			1
	Sets feet on the plantar support			2
**Pre-walk**	Up			1
	Up autonomous			4
	Down			1
	Down autonomous			4
	Warm up			1
	Step			1
	4 steps autonomous			5
	Directional changes			4
**Walk**	Easy-map 0.26 m	Slow		1
		Medium		1
		Fast		1
	Intermediate map 0.48 m	Slow		2
		Medium		3
		Fast		4
	Advanced 0.64 m	Slow		13
		Medium		13
		Fast		-
**6MWT**	Walked distance	Borg	Breaks	
	45.64 m	15	4	4
	60.42 m	15	-	5
	58.5 m	16	1	6
	64.27 m	15	-	7
	71.83 m	18	1	8
	75.84 m	15	1	9
	77.28 m	15	2	10
	76.8 m	13	-	11
	74.42 m	15	1	12
	84.87 m	15	-	13

## Data Availability

Not applicable.
